# Supplementary material for: Phosphorylation of myelin regulatory factor by PRKG2 mediates demyelination in Huntington's disease
Source: EMBO Rep. 2020 Apr 9;21(6):e49783. doi: 10.15252/embr.201949783 (PMC9336218; doi:10.15252/embr.201949783)
Supplement: Supplementary file 1 — Expanded View Figures PDF [file EMBR-21-e49783-s001.pdf]

Expanded View Figures

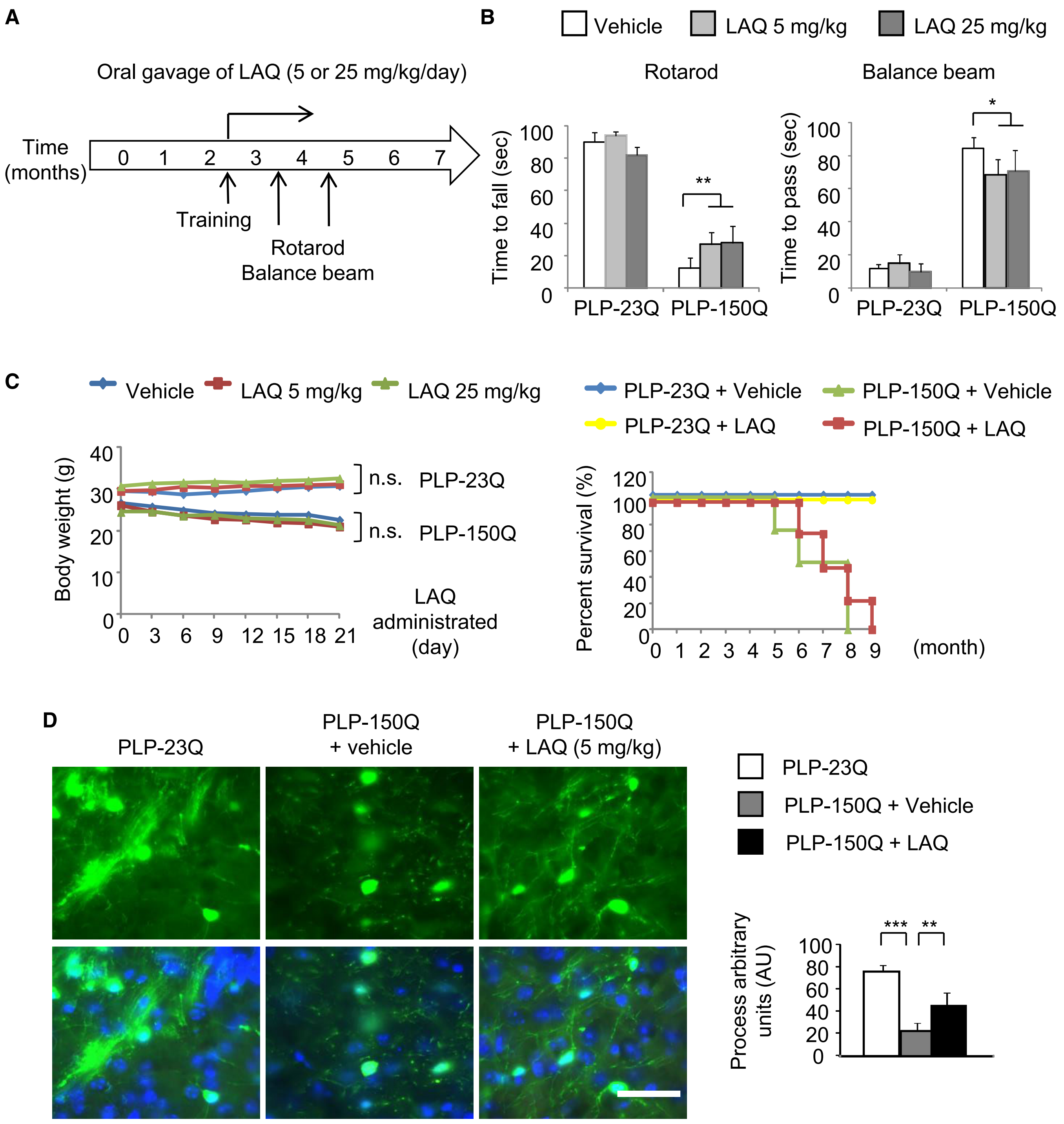

Figure EV1.

© 2020 The Authors

Figure EV1. (Related to Fig 1). LAQ partially alleviated phenotypes in PLP-150Q mice.

A Schematic of study design. PLP-150Q mice and the control PLP-23Q mice at the age of 3 months were orally administrated with LAQ (5 or 25 mg/kg/day as indicated in figure) for 2 months. The brain tissues were collected for pathological examination after behavioral studies.

B Rotarod performance and balance beam tests showed severe motor impairment in PLP-150Q mice. Administrating 5 or 25 mg/kg LAQ could alleviate the impairment. One-way ANOVA followed with Tukey's test. $^{**}P = 0.00735$; $^{*}P = 0.04086$; $n = 12$ mice per group for rotarod perform test and $n = 6$ mice per group for balance beam test. Data are presented as mean ± SEM.

C The PLP-150Q mice show reduced body weight and early death, which were not altered by the treatment with 5 or 25 mg/kg LAQ for 2 months. $n = 12$ mice each group, which were treated with LAQ at 3 months of age. Data are mean ± SEM.

D PLP-150Q/GFP mice show GFP labeling of oligodendrocyte processes, which are reduced in the corpus callosum when compared with PLP-23Q/GFP mice and were increased after 5 mg/kg LAQ treatment for 2 months. Quantitative analysis of the number of GFP-positive oligodendrocytes and process length was shown under the micrographs. $n = 3$ mice in each group. Process length was presented in a.u. One-way ANOVA followed with Tukey's test. $^{***}P = 3.92 \times 10^{-5}$; $^{**}P = 0.00187$. Data were mean ± SEM. Scale bar: 40 μm.

Figure EV2. (Related to Fig 1). Expression of the myelin-associated proteins in PLP-23Q mice treated with LAQ.

A Western blotting showing the multiple myelin proteins (MBP, MOBP, and MOG) in the corpus callosum in PLP-23Q mice that were treated with LAQ (5 or 25 mg/kg) at 3 months of age for 2 months. Vinculin served as a loading control.

B Quantitative PCR analysis of myelin-associated gene (MBP and MOG) in the corpus callosum of LAQ (5 or 25 mg/kg)-treated PLP-23Q mice. $n = 3$ each group. Data are mean ± SEM.

C Western blotting with anti-HTT (EM48) showing the aggregated and soluble huntingtin in the corpus callosum in PLP-150Q mice that were treated with LAQ (5 mg/kg) at 3 months of age for 2 months. Beta-actin served as a loading control.

D Co-transfection of N-terminal MYRF (nMYRF) with N-terminal HTT containing 150Q or 23Q in HEK293 cells and immunoprecipitation of nMYRF. More HTT-150Q binds nMYRF than HTT-23Q. Five μM LAQ treatment could decrease the immunoprecipitated HTT. The ratio of precipitated HTT to input in transfected cells treated with DMSO or LAQ obtained from three independent experiments was shown on the right. One-way ANOVA followed with Tukey's test. $^{***}P = 1.25 \times 10^{-5}$; $^{**}P = 0.00552$. Data are mean ± SEM.

© 2020 The Authors

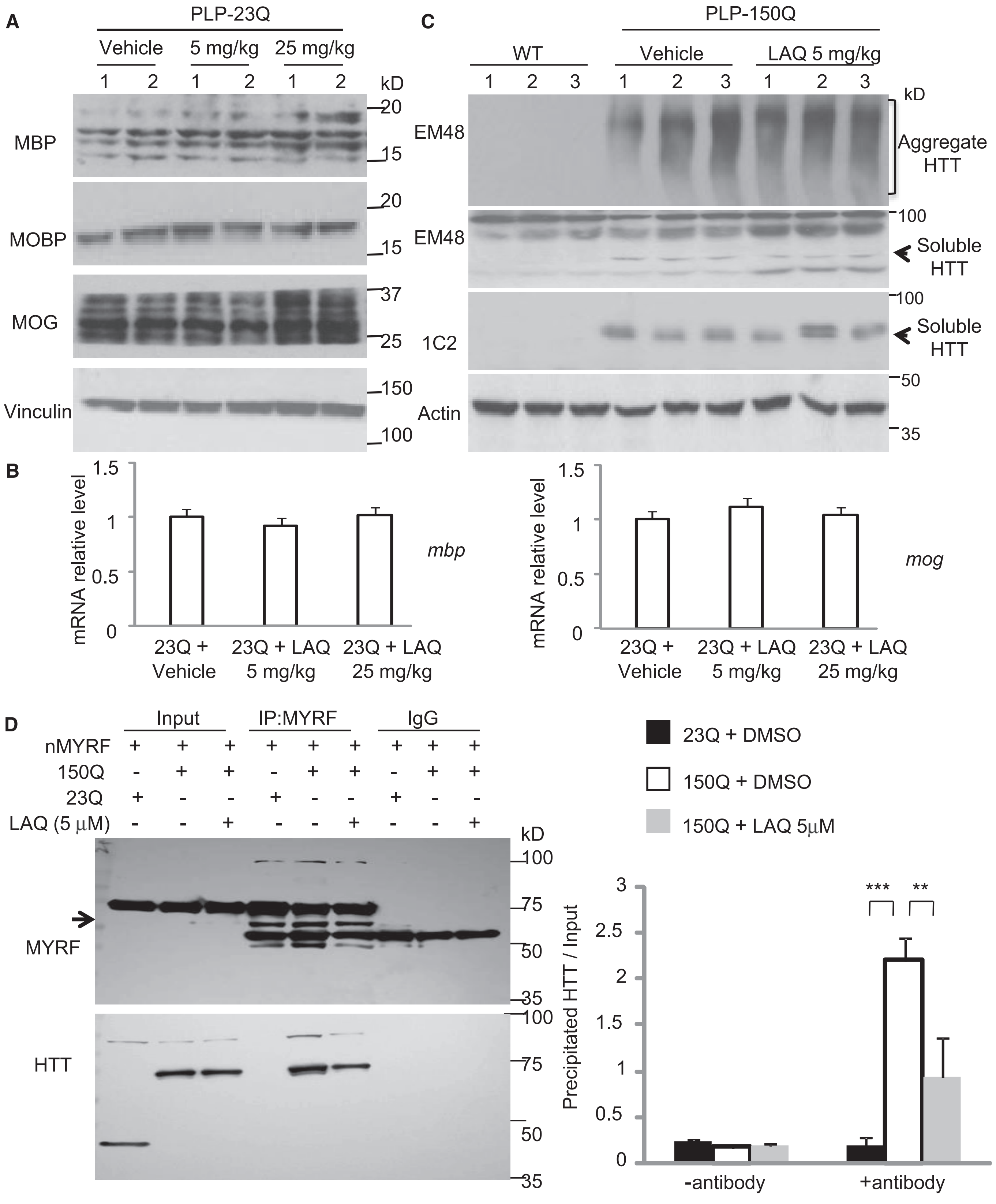

Figure EV2.

 © 2020 The Authors

Figure EV3. (Related to Figs 2 and 3). The anti-pMYRF (Ser259) antibody recognized phosphor-Serine 259 in N-terminal MYRF.

A Predicted potential phosphorylation sites in mouse N-terminal MYRF (nMYRF). S259 (red) is a top candidate.

B Mass spectrometry of the immunoprecipitated MYRF revealed an enrichment phosphorylated signal at pS259.

C The sequences of mutant MYRF S259A and S261A verified Ser259 substitution.

D Western blotting of HEK293 cell expressing wild-type N-terminal MYRF (nMYRF), S259A or S261A cDNAs, and mouse tissues showing that replacing Ser259 with alanine can eliminate the labeling of MYRF by antibody to Ser259. The samples were also probed with antibody to total MYRF (Sigma, HAP018310) and pre-immune serum. endo-fMYRF: endogenous full-length MYRF; endo-nMYRF: endogenous N-terminal MYRF; trans-MYRF: transfected MYRF.

E The anti-MYRF (Sigma, HAP018310) immunohistochemical staining of the corpus callosum of PLP-23Q and PLP-150Q mice showing no significant effect on the total MYRF expression by LAQ (5 mg/kg) treatment. n = 3 mice in each group. Scale bar: 10 μm. Quantitative analysis of the MYRF-positive cells per field (40×) was presented on the right. Data were mean ± SEM.

© 2020 The Authors

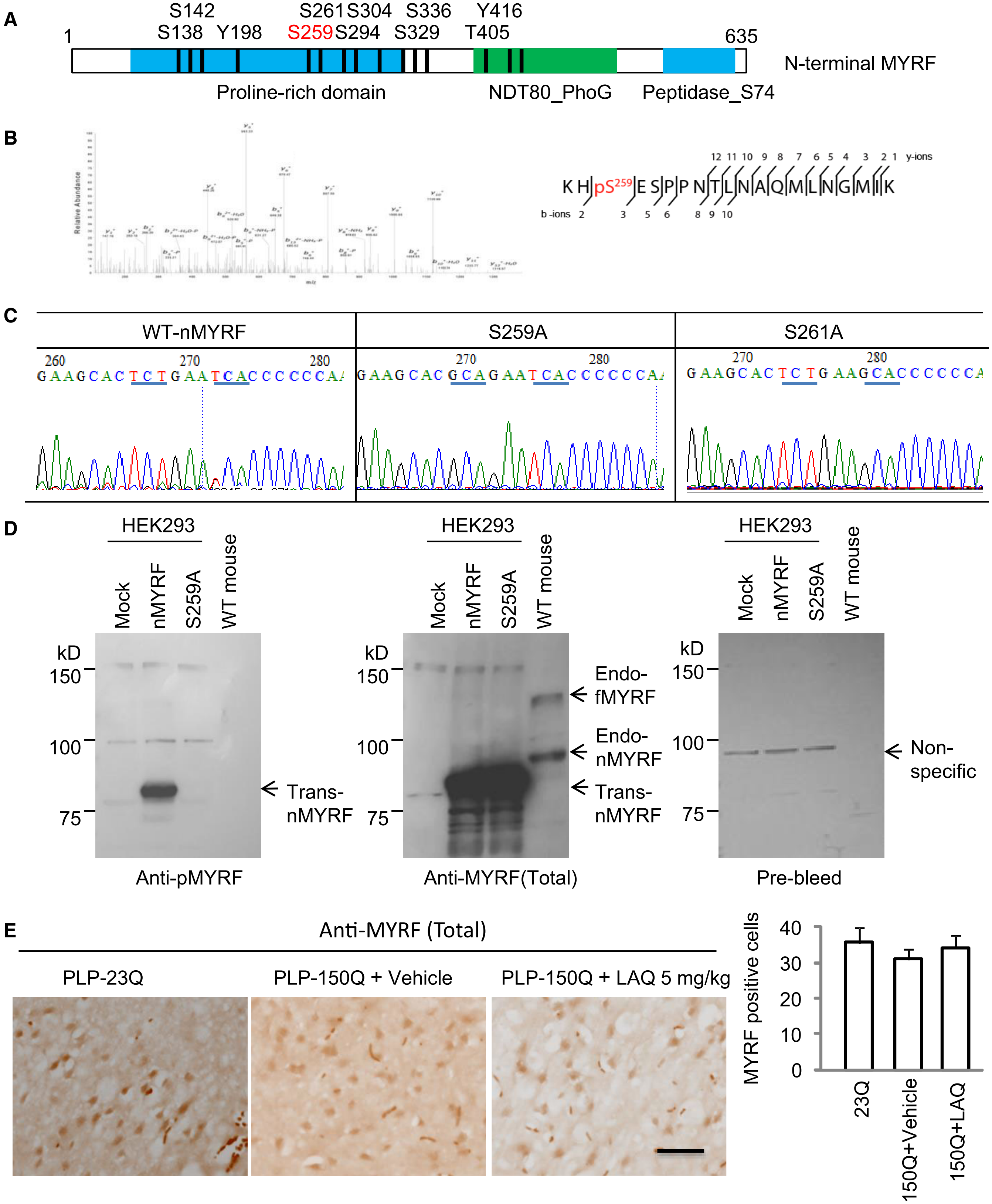

Figure EV3.

© 2020 The Authors

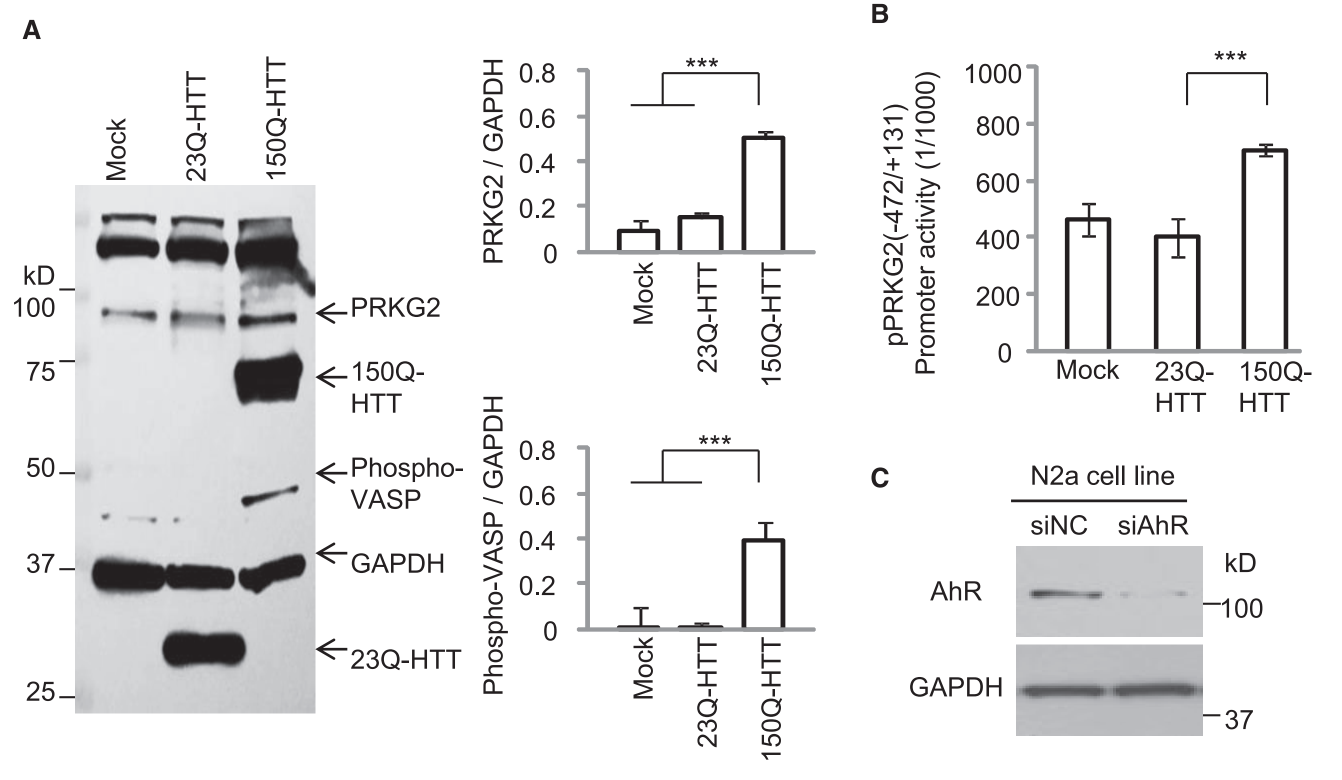

Figure EV4. (Related to Figs 4 and 5). Mutant HTT promotes PRKG2 gene transcript expression.

A N2a cells transfected with 23Q-HTT or 150Q-HTT were examined by the antibody of PRKG2 or phosphor-VASP (pVASP). Note that both the expression of PRKG2 and pVASP was increased by 150Q-HTT. GAPDH served as control. Ratios of PRKG2 or phosphorylated VASP (p-VASP) to GAPDH obtained from three independent experiments were presented on the right. One-way ANOVA followed with Tukey's test. PRKG2: $***P = 0.00032$; p-VASP: $***P = 0.00059$. Data are mean ± SEM.

B The co-transfection of 150Q-HTT with core promoter of PRKG2 (−472/+131) displayed an obvious induction of the transcription activity from three independent experiments, compared with the 23Q-HTT or Mock control. One-way ANOVA followed with Tukey's test. $***P < 0.001$. Data are mean ± SEM.

C Western blotting of transfected N2a cells showing that AhR siRNA, but not the scrambled control siRNA, treatment for 72 h inhibited AhR protein expression. The blots were probed with anti-AhR antibody.

Figure EV5. (Related to Figs 6 and 7). PRKG2 overexpression phosphorylates Ser259 in MYRF.

A Immunohistochemical staining with antibody to anti-Ser259 showed that the AAV-PRKG2 injection promoted MYRF phosphorylation in the corpus callosum (CC). Ctx: cortex, Str: striatum. Scale bar: 40 μm.

B The anti-MYRF (Sigma, HAP018310) immunohistochemical staining of the corpus callosum showing no significant effect on the total MYRF expression by AAV-PRKG2 injection. Scale bar: 100 μm.

C Western blotting of the corpus callosum region of PLP-150Q/Cas9 mice indicated the expression of both Cas9 and 150Q-HTT, compared with PLP-150Q, transgenic Cas9 mice, and WT mice. Mutant HTT was detected by 1C2 and EM48 antibody, and Cas9 was detected by anti-Cas9 antibody.

D Genome-editing activity of PRKG2-gRNA was tested using T7E1 assay. N2a cells were co-transfected with PRKG2-gRNA and Cas9 plasmids or transfected with PRKG2-gRNA plasmid alone as control. The T7E1 assay was carried out using PCR products of genome PRKG2 DNA from transfected N2a cells. The arrow indicates cleaved PCR products by T7E1 enzyme. Control is control-gRNA.

E Mutations in the CRISPR/Cas9 targeted PRKG2 gene were identified by TA cloning and subsequent sequencing.

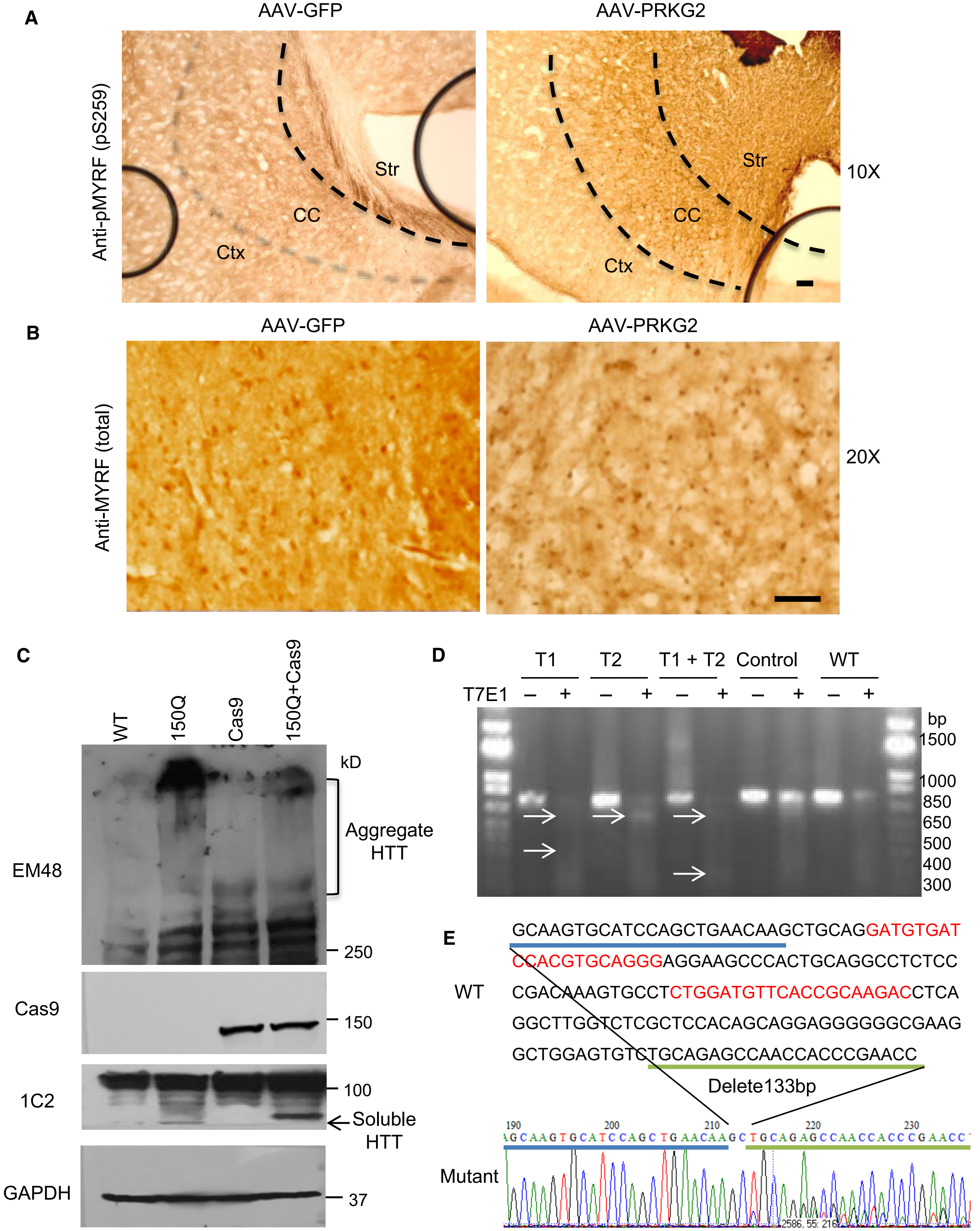

Figure EV5.

 © 2020 The Authors
